# Supplementary material for: Arctic Sea Ice Microalga Chlamydomonas latifrons KNF0041: Identification and Statistical Optimization of Medium for Enhanced Biomass and Omega-3/Omega-6
Source: Mar Drugs. 2023 Aug 17;21(8):454. doi: 10.3390/md21080454 (PMC10456082; doi:10.3390/md21080454)
Supplement: Supplementary file 1 [file marinedrugs-21-00454-s001.zip › Supplementary Table S1.docx]

Supplementary Table S1. Morphological comparison of strain KNF0041 and several related species.

|  | ***Chlamydomonas latifrons***  **(KNF0041)** | ***Chlamydomonas reinhardtii*** | ***Chlamydomonas incerta*** | ***Chlamydomonas schloesseri*** |
| --- | --- | --- | --- | --- |
| Cell shape | Spherical – broadly ellipsoid – broadly ovoid | Spherical – broadly ellipsoid – ovoid - obovoid | Spherical – broadly ellipsoid | Spherical – broadly ellipsoid – ovoid – obovoid |
| Cell length x cell width (μm) | 11.0–19.1  × 9.3–16.6 | 8.3–9.6  × 10.9–12.9 (12.6–15.4) | 8.0–9.5  × 9.7–12.4 (11.9–15.1) | 8.7–9.3  × 9.6–11.9 |
| Papilla | Yes | No | No | No |
| Mucilage surrounding the vegetative cells | - | - | - | + |
| Contractile vacuoles | 2 apical | 2 apical | 2 apical | 2 apical |
| Length of flagella | Slightly longer as the cell | Slightly longer as the cell | Slightly longer as the cell | Slightly longer as the cell |
| Position of the nucleus | Above the middle of the cell | Above the middle of the cell | Above the middle of the cell | Above the middle of the cell |
| Chloroplast shape | Cup-shaped | Cup-shaped, sometimes with small incisions | Cup-shaped | Cup-shaped, slightly lobed |
| Pyrenoid | Large, round to broadly ellipsoidal, in basal position | Small, round, in basal position | Medium, round-slightly ellipsoid, in basal position | Medium, round-slightly ellipsoid, in basal position |
| Eyespot | Elliptic in anterior position | Elliptic in anterior position | Elliptic in anterior position | Small elliptic in anterior position |
| Number of zoospores | 2–4 | 2–4–8–16 | 2–4 | 2–4–8 |
| Reference | This study | Pröschold et al. (2018) | Pröschold et al. (2018) | Pröschold et al. (2018) |
