# Supplementary figures and images for: Arctic Sea Ice Microalga Chlamydomonas latifrons KNF0041: Identification and Statistical Optimization of Medium for Enhanced Biomass and Omega-3/Omega-6
Source: Mar Drugs. 2023 Aug 17;21(8):454. doi: 10.3390/md21080454 (PMC10456082; doi:10.3390/md21080454)

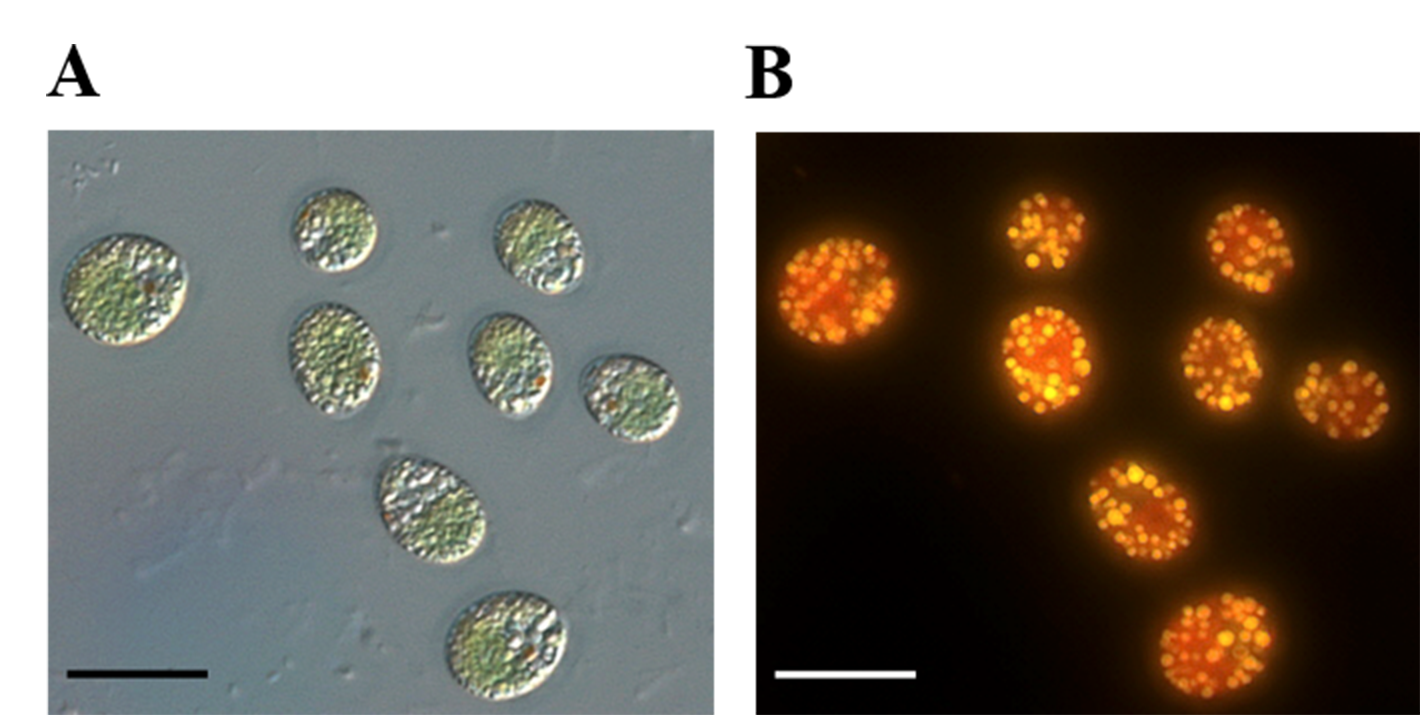

Supplement: Supplementary file 1 [file marinedrugs-21-00454-s001.zip › Supplementary Figure S1.png]

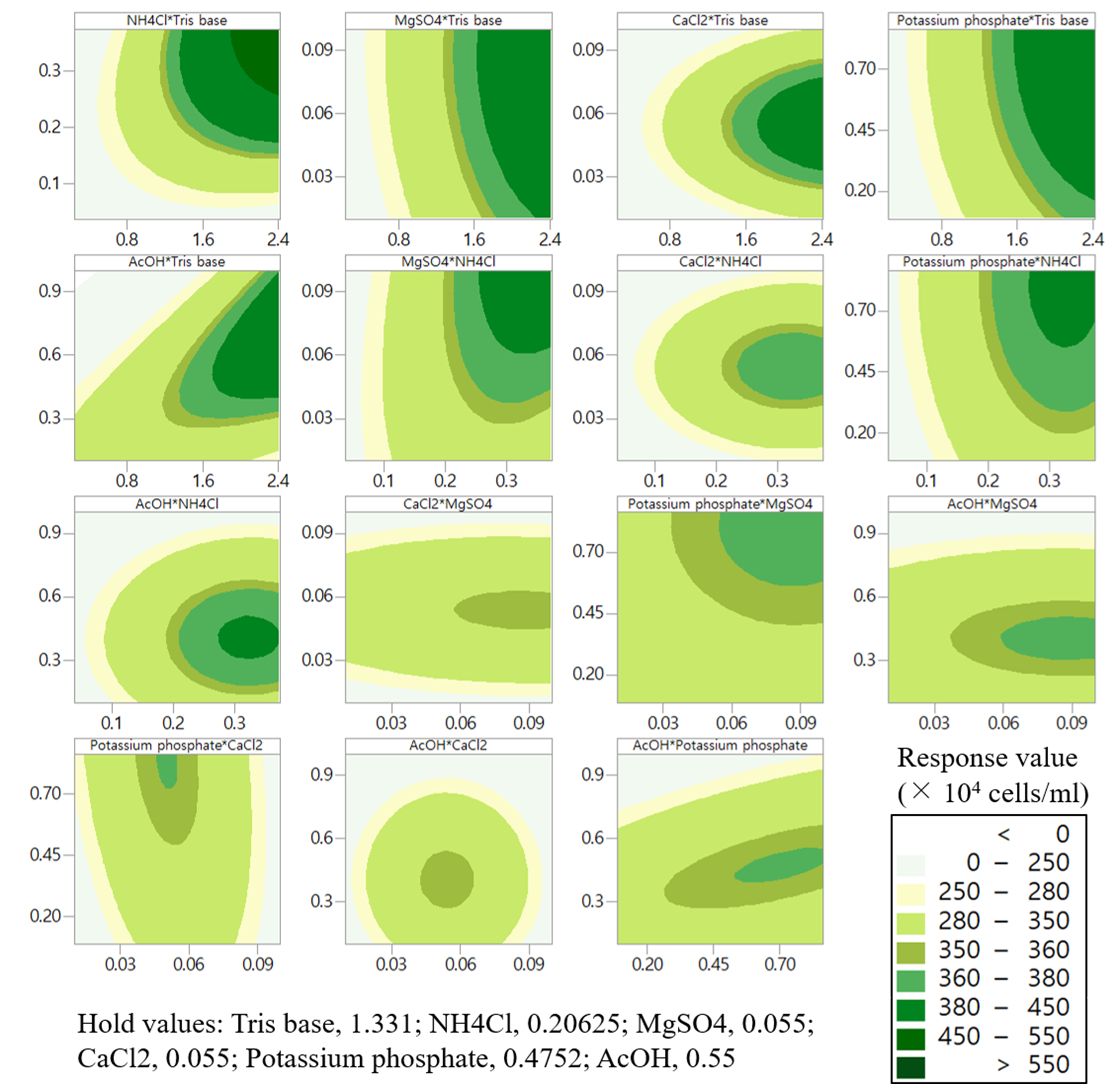

Supplement: Supplementary file 1 [file marinedrugs-21-00454-s001.zip › Supplementary Figure S2.png]

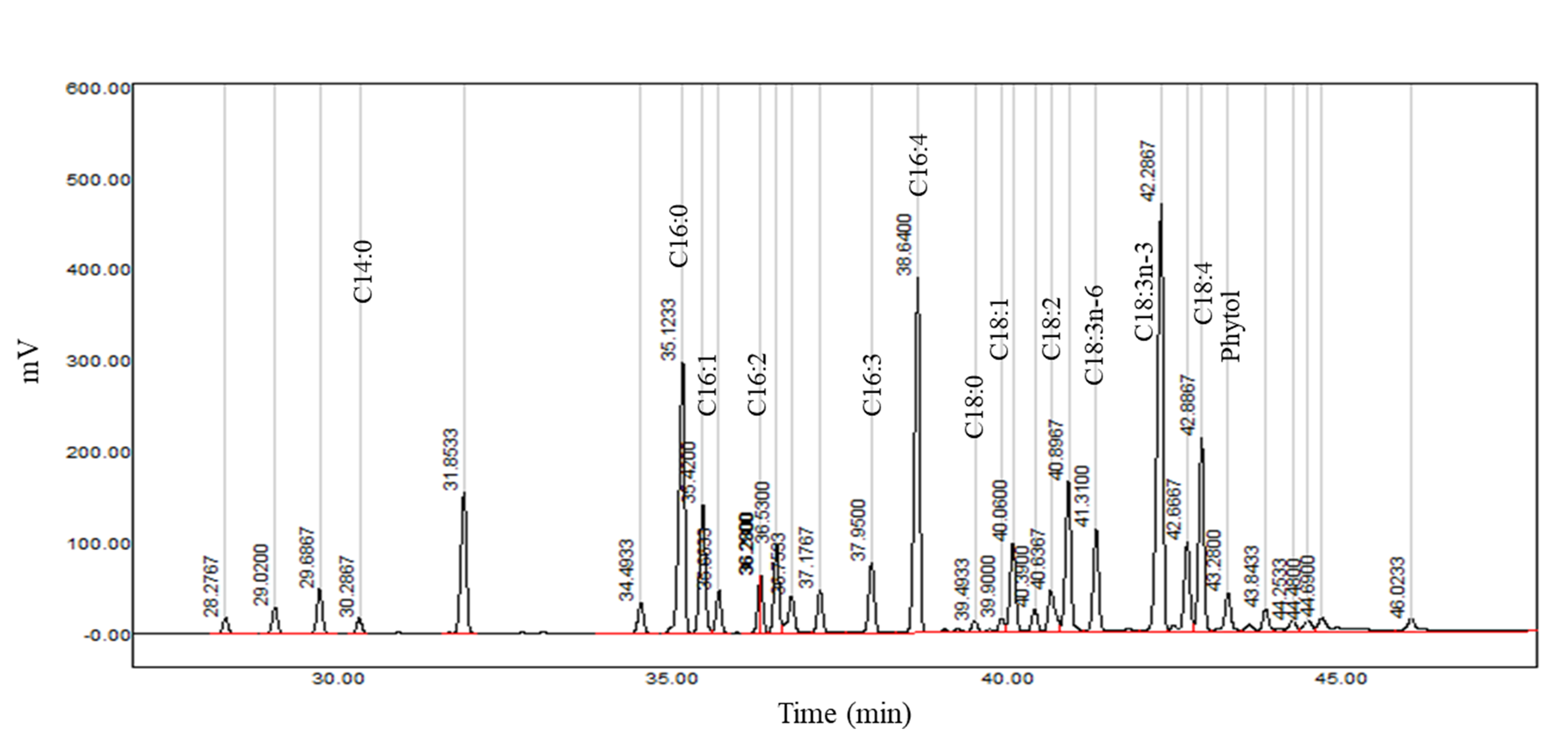

Supplement: Supplementary file 1 [file marinedrugs-21-00454-s001.zip › Supplementary Figure S3.png]
